# The appearance of phagocytic microglia in the postnatal brain of Niemann Pick type C mice is developmentally regulated and underscores shortfalls in fine odor discrimination

**DOI:** 10.1002/jcp.30909

**Published:** 2022-11-02

**Authors:** Alessandro Rava, Piergiorgio La Rosa, Giampiero Palladino, Jessica Dragotto, Antonio Totaro, Jessica Tiberi, Sonia Canterini, Sergio Oddi, Maria Teresa Fiorenza

**Affiliations:** 1 Division of Neuroscience, Department of Psychology, University LaSapienza, Rome, Italy; 2 PhD program in Behavioral Neuroscience, University La Sapienza, Rome, Italy; 3 European Center for Brain Research, IRCCS Fondazione Santa Lucia, Rome, Italy; 4 Faculty of Veterinary Medicine, University of Teramo, Teramo, Italy

**Keywords:** microglia, *Npc1*, olfaction assessment, phagocytosis1

## Abstract

The loss of NPC1 or NPC2 function results in cholesterol and sphingolipid dyshomeostasis that impairs developmental trajectories, predisposing the postnatal brain to the appearance of pathological signs, including progressive and stereotyped Purkinje cell loss and microgliosis. Despite increasing evidence reporting the activation of pro-inflammatory microglia as a cardinal event of NPC1 disease progression at symptomatic stages both in patients and preclinical models, how microglia cells respond to altered neurodevelopmental dynamics remains not completely understood. To gain an insight on this issue, we have characterized patterns of microglia activation in the early postnatal cerebellum and young adult olfactory bulb of the hypomorphic *Npc1^nmf164^* mouse model. Previous evidence has shown that both these areas display a number of anomalies affecting neuron and glial cell proliferation and differentiation, which largely anticipate cellular changes and clinical signs, raising our interest on how microglia interplay to these changes. Even so, to separate the contribution of cues provided by the dysfunctional microenvironment we have also studied microglia isolated from mice of increasing ages and cultured in vitro for 1 week. Our findings show that microglia of both cerebellum and olfactory bulb of *Npc1^nmf164^* mice adopt an activated phenotype, characterized by increased cell proliferation, enlarged soma size and de-ramified processes, as well as a robust phagocytic activity, in a time-and space-specific manner. Enhanced phagocytosis associates with a profound remodeling of gene expression signatures towards gene products involved in chemotaxis, cell recognition and engulfment, including Cd68 and Trem2. These early changes in microglia morphology and activities are induced by region-specific developmental anomalies that likely anticipate alterations in neuronal connectivity. As a proof of concept, we show that microglia activation within the granule cell layer and glomerular layer of the olfactory bulb of *Npc1^nmf164^* mice is associated with shortfalls in fine odor discrimination.

## 1 Introduction

Microglia are brain immune cells playing an active and indispensable contribution to proper CNS development and function (Thion & Garel, 2020). Processes modulated by microglia include embryonic and postnatal neurogenesis, synaptic formation and elimination, axonal growth, myelinogenesis, neuronal survival and wiring (Sato, 2015; Thion & Garel, 2020; Zengeler & Lukens, 2021). Furthermore, microglia mediate the turnover of neuronal precursors by removing the excess of newly generated neurons and promoting the apoptosis of developing and differentiated cells (Arandjelovic & Ravichandran et al., 2015; Sato, 2015).

CNS microglia originate from myeloid precursors, which seed the embryonic developing brain where they expand into a population that self-renews throughout life and differentiates according to both intrinsic and local factors (Thion & Garel, 2020). This circumstance positions microglial cells in close vicinity to developing neurons largely before astrocytes and oligodendrocytes are generated, favoring a symbiotic relationship and making the differentiation of microglia and maturing neurons profoundly interdependent (Szepesi et al., 2018; Thion & Garel, 2020). On the other hand, the intimate microglia–neuron intertwining raises the question as to whether microglia is involved in the initiation and/or progression of neurodevelopmental disorders (Zengeler & Lukens, 2021). Indeed, highly plastic microglial cells promptly respond to a variety of genetic and environmental factors by switching to appropriate and adaptive activation states aimed at maintaining tissue homeostasis, thus acting as excellent sentinels of milieu variation (Paolicelli & Ferretti,2017). However, whether microglia response is maladaptive, insufficient or exaggerated, it may induce aberrant or dysfunctional circuit formation (Paolicelli & Ferretti, 2017; Zengeler & Lukens, 2021).

These observations are particularly interesting in the context of Niemann-Pick type C (NPC) disease progression. NPC disease is a rare inherited neurodegenerative disorder characterized by massive lysosomal cholesterol and sphingolipid accumulation caused by genetic loss-of-function mutations of the endolysosomal lipid exporters NPC1 (95% of clinical cases) or NPC2 (Vanier, 2015). Dysfunctional lipid export from lysosomes disrupts intracellular lipid homeostasis, altering developmental trajectories and predisposing to the appearance of overt pathological signs in Npc1-deficient mouse models, including a progressive and stereotyped Purkinje cell loss and microgliosis (Baudry et al., 2003; Fiorenza et al., 2022; Martin et al., 2019; Pressey et al., 2012). However, while pro-inflammatory microglia activation, at symptomatic stages typically occurs both in patients and preclinical models (Cologna et al., 2014; Colombo et al., 2021; Cougnoux et al., 2018; Pressey et al., 2012), how microglia cells respond to altered neurodevelopmental dynamics remains not completely understood (Boyle et al., 2020; Colombo et al., 2021).

Based on this premise, we thought that the developing cerebellum of NPC1 mouse models was a suitable model system for studying how microglia interplays with the significant number of developmental alterations of both neurons and glial cells displayed by these mice (Canterini et al., 2017; Caporali et al., 2016; Kavetsky et al., 2019; Nusca et al., 2014; Oddi et al., 2019). We found that the microglial morphological and biochemical profile undergoes significant morpho-functional remodeling in the various subfields of the cerebellum starting from the second postnatal week of age.

In light of these findings, we have extended our analysis to the olfactory bulb (OB) based on the following reasoning. First, the OB is the site of intense neuronal turnover and synaptic reorganization due to the continued replacement of local granule interneurons and olfactory sensory neurons from precursor cells throughout life (Lledo et al., 2005). Second, the production of postnatal-born neuroblasts in the SVZ is reduced and their integration in the granule cell layer (GCL) of the OB is defective in young-adult Npc1-deficient mice, indicating that Npc1 loss of function affects the maintenance of olfactory neuronal population (Dragotto et al., 2019; Seo et al., 2014; Seo et al., 2018). These defects are clearly seen at 4 weeks of age in knockout *Npc1*-/- mice (Seo et al., 2014), when there is no overt manifestation of disease signs, yet, whereas a significant decrease of SVZ neuroblasts is observed starting from P60 in *Npc1^nmf164/nmf164^* mice (Dragotto et al., 2019).

Our results show a significant increase of activated microglia in the GCL and glomerular layer (GL) of young adult *Npc1^nmf164/nmf164^* mouse OB. Activated microglia are engaged in phagocytic activity, which reasonably represents an indirect sign of altered differentiation of newly generated neurons. This possibility is strengthened by the presence of shortfalls in fine odor discrimination ability, which anticipates major olfactory dysfunction of NPC disease mouse model (Dragotto et al., 2019; Seo et al., 2014).

Altogether our findings support the view that early microglia activation of Npc1-deficient mouse brain emerges as a response to local perturbations of developmental trajectories in a time-/brain area-dependent manner. This view is supported by evidence that the differentiation of various cerebellar neural precursors is actually affected by Npc1 deficiency during the 2nd and 3rd postnatal week (reviewed in Fiorenza et al., 2022). Although a thorough characterization of cellular anomalies of the OB is not available, yet, the intracellular cholesterol defect caused by Npc1 deficiency is expected to similarly affect this area, by dysregulating mechanisms fundamental for neural progenitor proliferation, differentiation, and synaptic refinement.

## 2 Material And Methods

### 2.1 Animals and husbandry

*Npc1^nmf164/nmf164^* mice, hereafter named *Npc1^nmf164^*, with BALB/cJ background, obtained from heterozygous crosses were exposed to a 12 h light-dark cycle and received food and water ad libitum. Pup genotypes were identified by PCR analysis of tail DNA (Maue et al., 2012). Sex and age-matched littermates were group-housed in standard cages (Size: 13 cm height × 26 cm length × 20 width) enriched with a transparent red polycarbonate igloo house.

Experimental protocols were approved by the Italian Ministry of Health, and experiments were conducted according to the ethical and safety rules and guidelines for the use of animals in biomedical research provided by the relevant Italian laws and European Union's directives (Italian Legislative Decree 26/2014 and 2010/63/EU). Wild-type (*wt*) littermates were used as controls in all experiments.

### 2.2 Histology and immunoreactions

To obtain brains for histological analyses, deeply anesthetized animals were transcardially perfused with 4% PFA in PBS. Upon dissection from the skull, brains were post-fixed overnight in 4% PFA and after several washes with PBS they were soaked in 30% sucrose for cryoprotection. Specimens were embedded in FSC22 medium (Leica Biosystem) and cut into 35 μm thick cryostat sections (Leica Biosystem). Free-floating sagittal slices were rinsed with 0.1 M PBS and incubated with 3% hydrogen peroxide (Sigma-Aldrich) for 30 min at room temperature (RT) to inactivate endogenous peroxidase. Free-floating sections were then incubated with blocking-permeabilization solution, PBS supplemented with 1% bovine serum albumin (BSA) and 0.3% Triton X-100, for 2 h at RT and immunostained with primary antibody against Iba1 (1:500 in blocking solution; Wako Chemicals) for 72 h at 4°C with constant shaking. Subsequently, sections were rinsed in PBS and washed three times for 10 min in PBST (PBS supplemented with 0.3% Triton X-100). Iba1-positive cells were visualized using the ABC Elite Kit with 3,3'-Diaminobenzidine (DAB) as chromogen (Vector Laboratories), according to manufacturer's protocol. Finally, sections were rinsed in PBS, mounted on glass slides (Leica Biosystems), cleared with ascending alcohol concentrations, defatted with xylene and cover-slipped using Eukitt mounting medium.

The secondary antibody used to detect Iba1-positive cells by immunofluorescence was the Alexa Fluor 555-conjugated (Invitrogen, Milan, Italy Sigma, 1:800 dilution in PBS-BSA, for 2 h at RT). Nuclei were counterstained with Hoechst 33258 (Sigma-Aldrich, 1:1000). Immunoreaction specificity was assessed by omitting the primary antibody both in IHC and IF assays.

### 2.3 Nissl staining

For Nissl staining, cryostat sections were mounted on slides and air-dried for 24–48 h at RT. Subsequently, sections were hydrated with a series of decreasing concentrations of ethanol (100%, 95%, 70%, and 50%) for 2 min each, and rinsed in bi-distilled water (bdH_2_0) for 2 min. Then, sections were stained with 0.1%cresyl violet solution, rapidly washed with bdH_2_O for 1 min and dehydrated in a series of graded alcohols (70%, 95%, 100% 2x) for 2 min each. Finally, sections were clarified in xylene for 5 min and cover-slipped using Eukitt mounting medium.

### 2.4 Microglia assessment and cell count

A Leica DM500 microscope equipped with high-resolution digital camera (Leica MC120 HD) and MBF software was used for image acquisition. The number of Iba1-positive cells was determined in regions of interest (ROI) of 500 μm^2^ (2–4 sections/mice), including the cerebellar white matter (WM), internal granule layer (IGL), Purkinje cell layer (PCL) and molecular layer (ML), both for anterior (II–III) and posterior lobules (IX–X). Microglia were also classified as ramified, hypertrophic, bushy or ameboid based on their morphologies (Martini et al., 2020; Savage et al., 2019): (1) Ramified—small round soma and extended, highly branched processes; (2) Hypertrophic—larger soma and thicker, shorter and less branched processes; (3) Bushy - swollen and enlarged soma, and an excess of thicker, shorter processes with few branches; (4) Stout/Ameboid - retracted processes and irregular shape.

The quantification of Iba1-positive cells of the olfactory bulb was performed by outlining 500 μm^2^ ROI that included the GCL. Cells and phagocytic cups were manually counted on acquired images at 20X magnification, by using the cell counter plugin from the ImageJ/Fiji NIH software (Version 1.51, National Institutes of Health).

### 2.5 Morphological 3D reconstruction

For 3D reconstruction, individual microglia cells were imaged using the NeuroLucida image analysis system (MBF, Bioscience) connected to an Olympus BX53 microscope (100X/1.25 numerical aperture). The depth of the z-plane was varied to ensure optimal clarity for an accurate and precise morphological 3D reconstruction. Iba-1 positive cells were selected randomly and analyzed for morphometric parameters describing the shape and spatial structure of each individual microglial cell, as previously described (Kalambogias et al., 2020). In detail, the following parameters were analyzed:

(1) Soma perimeter, the outline length of a given object expressed in microns;
(2) Soma area, the total number of microns present in a single object;
(3) Feret max, maximum diameter of soma;
(4) Feret min, minimum diameter perpendicular to the feret max;
(5) Aspect ratio, feret max/feret min. An aspect ratio closer to 1, indicates a more symmetric soma;
(6) Form factor, (4π × area)/perimeter^2^, directly reflects the complexity of the somatic perimeter;
(7) Compactness, [√4/π) × area]/feret max;
(8) Roundness, (4 × area)/(π × feret max^2^);
(9) Convexity, (convex contour)/perimeter);
(10) Solidity, the ratio of soma area as a whole over convex area.

Moreover, the following descriptors of branching pattern were analyzed: (1) total processes; (2) total nodes; (3) total ends; (4) total process length; (5) mean process length; (6) total process surface; (7) mean process surface; (8) total process volume; (9) mean process volume.

To further study the process complexity, we also performed a Sholl analysis of 3D reconstructed microglia cells by analyzing the number of intersections crossing concentric spheres as functions of soma distance. The starting radius was 5 μm with subsequent radius increments of 5 μm.

### 2.6 Phagocytic cup size analysis

The determination of phagocytic cup size was performed on the same IGL regions used for cell count by Neurolucida MBF software. Single cups were imaged with a 40x objective and max feret diameter was measured. This analysis was performed on 3 *wt* (Tot. 28 phagocytic cups) and 3 *Npc1^nmf164^* mice (Tot. 32 phagocytic cups).

### 2.7 Western blot analysis

Brains were rapidly removed from the skull, and cerebella were collected and stored at -80°C until use. Total proteins were extracted with RIPA buffer supplemented with protease inhibitor cocktail solution (cat. sc-24948, Santa Cruz Biotechnology), and their concentration was routinely determined by Bradford's colorimetric assay. Equal amounts of total protein/lane (45 μg/lane) were subjected to SDS-PAGE under reducing conditions. Subsequently, gels were electroblotted onto nitrocellulose (Whatman, Springfield Mill) and a Ponceau's red staining was performed to verify the protein transfer efficiency and loading amount. Membranes were blocked with 2% powdered milk (GE Healthcare) in TBST (Tris-buffered saline with 0.05% Tween-20) for 1 h to RT and immunoreacted with the following primary antibodies: mouse anti-Actin (1:1000, Sigma Aldrich, cat. no. A-5441); rabbit anti-Iba1 (1:500, cat. E-AB-60328, Elabscience); rabbit anti-Trem2 (1:200, cat. E-AB-16947, Elabscience); rabbit anti-Caspase3 (1:200, cat. HPA002643, SIGMA life science) and rabbit anti-Cleaved-caspase3 (1:1000, Cat. 9661, Cell signaling). Following three washings with TBS supplemented with 0.05% Tween-20 (TBST) per 3 min, membranes were incubated with the appropriate horseradish peroxidase-conjugated secondary antibody for 1 h (1:5000; Santa Cruz Biotechnology). After three washings withTBST per 3 min, followed by 1 washing inTBS (pH 7.4), membranes were processed for chemiluminescence detection (Luminata Crescendo Western HRP substrate, Millipore, Burlington), according to the manufacturer's instructions. Chemiluminescence signals were detected in a CDiGit blot scanner (LI-COR) and analyzed by Image Studio Software 4.0.21 (LI-COR). Densities of protein bands were reported as ratios between the protein of interest and the Ponceau's red staining (Sander et al., 2019). Similar results were obtained when the beta-actin band intensity was used as reference.

### 2.8 RNA isolation and RT-qPCR

Total RNA isolation from brain tissue and cultured cells was performed by using NucleoSpin RNA plus kit (cat. 740984.5, Macherey-Nagel). RNA was retrotranscribed using Oligo(dt) and One script Plus Reverse transcriptase reagents (cat. G236, ABM) in a 15 min at the 50°C reaction, followed by a stop reaction step at the 85°C for 5 min. Three/two replicas of 0.5 μl of cDNA were amplified using Power Up SYBR Green PCR Master Mix (Thermo Fisher, Scientific) for tissue or cell culture experiments in Quantum 3 Real-Time PCR Detection System (Applied Biosystems). Primer sequences were: Trem2, F: GCCTTCCT GAAGAAGCGGAA; R: GAGTGATGGTGACGGTTCCA; Cd68, F: ACTTC GGGCCATGTTTCTCT;R:GCTGGTAGGTTGATTGTCGT;Cd11b,F:GTG TGACTACAGCACAAGCCG; R: CCCAAGGACATATTCACAGCCT; CX3 CR1 F: CACCATTAGTCTGGGCGTCT; R:GATGCGGAAGTAGCAAAAGC; CX3CL1 F: TGAGAGTGAGGAAGCCAACC; R:GGAACCAACAAAGTC CGATG S16, F: AGGAGCGATTTGCTGGTGTGG; R: GCTACCAGGG CCTTTGAGATGGA, and were validated by using PrimerBlast (NIH), and their specificity was assessed by melting curves and amplified product electrophoresis in 2% agarose gels. A typical amplification protocol was 50°C for 2 min, 95°C for 2 min, 95°C for 15 s, and 40 cycles at 60°C for 30 s. S16 was used as a housekeeping reference gene. Gene expression was analyzed using the 2^-ΔΔCt^ method.

### 2.9 Isolation and in vitro culture of primary microglia from pup and adult mouse brains

Whole brains from *wt* and *Npc1^nmf164^* pups (P6) and young adult (P30) mice were dissected and enzymatically dissociated in a single cell-suspension by using the neural tissue dissociation kit (cat. 130-092-628 and 130-107-677, Miltenyi Biotec, respectively). When adult brains were processed, myelinated components and other cell debris were carefully removed to facilitate the subsequent extraction steps. To positively select microglia, the cell suspension was incubated with CD11b-coniugated microbeads (cat. 130-093-634, Miltenyi Biotec) and pulled down using a MACS separation column (MS, cat. 130-04-201, Miltenyi Biotec). CD11b-positive mouse microglia were cultured in DMEM culture medium supplemented with 10% heat inactivated FBS for 7 days at 37°C and 5% CO2, and subsequently immunostained or harvested for RNA isolation.

### 2.10 Assessment of odor detection and discrimination

The odor detection ability was determined by using a typical habituation/dishabituation test (Arbuckle et al., 2015; M. Yang & Crawley, 2009) with slight modifications Briefly, on the test day, each mouse was habituated for 1 h to a bedding-supplied home cage (30 × 15 × 15 cm). Then, a dry cotton-tipped swab was fixed to the cage lid leaning down to the cage space for 30 min. This procedure is essential to reduce novelty-induced exploratory activity during the olfaction test. At the end of this habituation phase, the animal was exposed to a sequence of five odors delivered through cotton swabs, in the following sequence: distilled water, (2) vanilla (McCormick, Hunt Valley, MD, 1:100 dilution), (3) orange extract (McCormick, Hunt Valley, MD, 1:100 dilution), social odor 1 (social 1) and social odor 2 (social 2). Vanilla and orange extracts were selected because they are distinct, yet neutral odors; social odors consisted of urine samples from unfamiliar mice of the opposite (social 1) or same sex (social 2). For every exposure, the cotton swab was freshly prepared with a fixed amount of solution (5 and 4 μl for nonsocial and social odors, respectively). The test was video-recorded by an observer blind to the mouse genotype and exploratory activity determined as the time length during which the mouse nose was in contact with the cotton swab, or directed toward it at a distance ≤2 cm.

An experimental setting similar to that of habituation/dish-abituation test was exploited to determine fine olfactory discrimination performance using odorants that share a highly similar chemical structure and physicochemical properties (Abraham et al., 2010). In detail, two pairs of odorants were selected:

(i) (+)-Limonene and (–)-Limonene enantiomers, which smell like orange and lemon, respectively; and ii) butanol and pentanol alcohols, which differ for one Carbon atom only. Owing that paraffin mineral oil was used to dilute the odorants, animals were pretrained with plain mineral oil-laced cotton swabs for four presentations (60 s each, 2 min intervals) to ensure that subsequent exposure to an odorant-laced cotton swab did not elicit a response due to novelty. Next, mice were challenged with four sequential 60 s presentations of one odor, either (+)-Limonene or butanol, and then exposed to the respective structurally similar molecule, that is, (–)-Limonene or pentanol, for 60 s. The exploration time at each presentation was determined as for the habituation/dishabituation test. (+)-Limonene, (–)-Limonene, butanol, pentanol, and paraffin mineral oil were purchased from Sigma Aldrich, Milano, Italy.

### 2.11 Statistical analysis

Statistical analyses were performed with GraphPad Prism 8.0 software. All values are shown as the mean ± SEM. Experimental group size was as follows: *n* = 3–5 *wt* and *n* = 3–6 *Npc1^nmf164^* mice/ age for histology experiments, *n* = 3–6 *wt* and *n* = 3–7 *Npc1^nmf164^* mice/age for biochemical experiments, *n* = 3–4 *wt* and *n* = 3 *Npc1^nmf164^* mice/age for in vitro assays, *n* = 10 *wt* and *n* = 10 *Npc1^nmf164^* mice/age for olfaction assessment. Individual comparisons between two experimental groups were performed using the unpaired two-tailed *t*-test with Welch correction. For behavioral assessments, one-way analysis of variance (ANOVA) followed by post hoc tests was used. *p*-value of <0.05 was used as the criterion for significance. Statistical details (test used, sample size, *p*-value) can be also found in figure legends.

## 3 Results

### 3.1 Spatio-temporal distribution of microglia in the early postnatal cerebellum of *Npc1^nmf164^* mice

**Figure 1:**
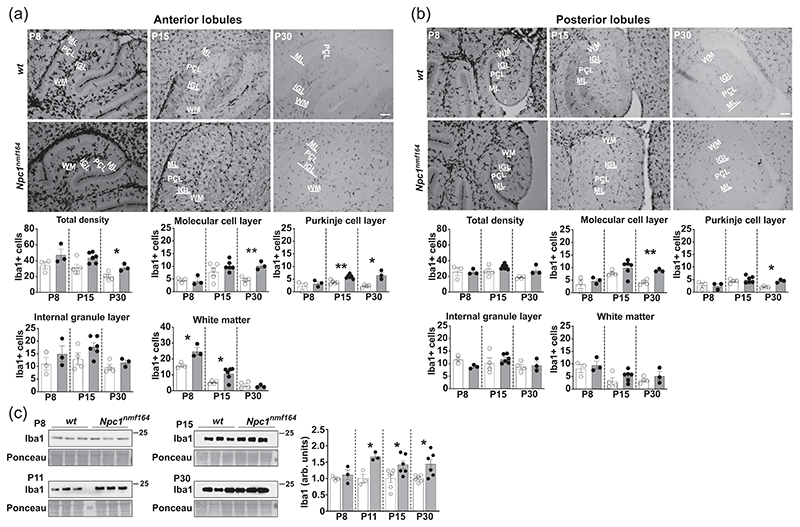
Changes of microglial distribution in the developing cerebellum of *Npc1^nmf164^* mice. (a, b) Representative images (upper panels) and quantitative analysis (bottom graphs) of Iba1-positive microglia in anterior (a) and posterior (b) cerebellar lobules of *wt* and *Npc1^nmf164^* mice of increasing age. Quantification was performed by counting Iba1-positive cells per ROI (500 μm^2^). Scale bars, 100 μm. IGL, internal granule cell layer; ML, molecular layer; PCL, purkinje cell layer; WM, white matter (Welch *t*-test, *p < 0.05, **<0.01; *n* = 3–5 *w*t, 3–6 *Npc1^nmf164^* mice/age). (c) Western blot analysis (left and center panels) and relative quantification (bars on the right; empty bars: *wt*; gray filled bars: *Npc1^nmf164^*) of Iba1 protein expression in the cerebellum of *wt* and *Npc1^nmf164^* of P8, P11, P15, and P30 mice. Densitometric values were normalized to the total protein content. Data are presented as mean ± SEM (Welch *t*-test, *p < 0.05, **<0.01; *n* = 3–6 *w*t, 3–6 *Npc1^nmf164^* mice/age).

Maintaining an appropriate number of microglia within the CNS is vital for tissue homeostasis since it secures the proper neuronal circuitry development and maturation (Thion & Garel, 2020). Previous studies have shown that microglia cells invade cerebellar cortical layers migrating from white matter (Nakayama et al., 2018), wherein the proliferation of microglia precursors peaks at the end of the first week after birth in mice (Li et al., 2019). To investigate the presence of possible anomalies of density and spatio-temporal distribution of microglia in the postnatal developing cerebellum of *Npc1^nmf164^* mice, in a first set of experiments we identified microglial cells by immunohistochemistry of cerebellar sections of post-natal Day 8 (P8), P15 and P30 mice with antibodies against the ionized calcium-binding adapter molecule 1 (Iba1), a specific microglia marker (Ito et al., 1998). This analysis showed a sharp increase of total microglia cell number starting from P8 in the anterior cerebellar lobules of the *Npc1^nmf164^* mice compared to age-matched *wt* mice (Figure 1a, total density).

When the spatiotemporal distribution of microglia in the various cerebellar layers was analyzed, microglia density was found particularly pronounced in the anterior lobule white matter of P8 *Npc1^nmf164^* mice compared to age-matched *wt*. Subsequently, the increase of microglial cells encompassed the entire cerebellar cortex at P15 and in the young adult mice, P30 (Figure 1a). Noteworthy, the rise of microglial cells appeared to be less pronounced in the posterior lobules, which displayed an increase of Iba1-positive cell number at P30 but not at earlier ages (Figure 1b). This anterior-posterior heterogeneity is even more evident in the cerebellum of P60 *Npc1^nmf164^* mice (Supporting Information: Figure S1). The increased density of Iba1-positive cells was confirmed by Western blot analysis of cerebellum total extracts, observing that the Iba1 expression level was significantly enhanced in Npc1-deficient mice compared to age-matched controls, from the second postnatal week of age. In particular, the Iba1 content was found to significantly increase at P11, consistently with the sharp microglia density increase observed at P8 already (Figure 1c).

To further characterize microglia changes in terms of differentiation and functional condition, we next classified Iba1-positive microglia as resting/surveillant or activated on the basis of their morphological features (see Section 2). Our analysis revealed no significant differences in the microglia morphology between *wt* and *Npc1^nmf164^* mice neither in anterior nor in posterior lobules at P8. However, as development proceeded, an increasing number of *wt* microglia acquired a resting/surveillant phenotype, characterized by a ramified morphology (Supporting Information: Figure S2A,B), whereas the fraction of ramified microglia of *Npc1^nmf164^* mice decreased starting from P15 and was accompanied by the appearance of microglia displaying hypertrophic/bushy or ameboid-like morphology with higher frequency, both in the anterior and posterior regions of cerebellum. This morphology typically recalls an activated state, which is often associated with increased phago-lysosomal activity (Zhao et al., 2018).

### 3.2 Microglia of P15 *Npc1^nmf164^* mouse cerebellum has morphological and molecular signatures of phagocytic activity

**Figure 2:**
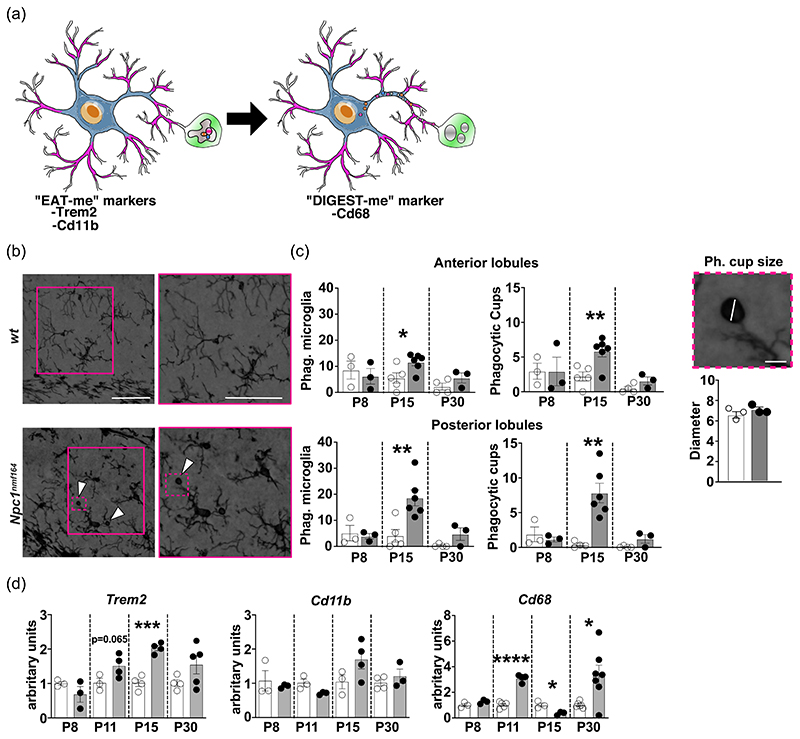
Microglia phagocytosis increases in the postnatal developing cerebellum of *Npc1^nmf164^* mice. (a) A scheme of phagocytic microglia. (b) Representative images (left panels) and magnification (right panels) of wt and *Npc1^nmf164^* mouse microglia detected by Iba1 antibody immunoreactivity. Insets outline nonphagocytic microglia (*wt* panels) and phagocytic microglia (*Npc1^nmf164^* panels), with arrowheads indicating phagocytic cups. Scale bar: 50 μm. (c, left to right) Bars indicate the fraction of phagocytic microglia cells and total number of phagocytic cups per ROI (500 μm^2^) in the anterior and posterior cerebellar lobules of P8, P15, and P30 mice (*n* = 3–5 *wt*, 3–6 *Npc1^nmf164^* mice/age; empty bars: wt; gray filled bars: *Npc1^nmf164^*). Higher magnification of a representative phagocytic cup; scale bar: 8 μm. Bars show max feret diameter of microglia phagocytic cups in the cerebellar IGL of P15 *wt* and *Npc1^nmf164^* mice (*n* = 3 *wt*, 3 *Npc1^nmf164^* mice). (d) RT-qPCR analysis of Trem2, Cd11b, and Cd68 transcript level expression performed on whole cerebella collected from *wt* and *Npc1^nmf164^* mice at increasing time points (P8-P30). S16 expression was used for normalization. Data are presented as mean ± SEM (Welch *t*-test, *p < 0.05, **<0.01; *n* = 3–6 *wt*, 3–7 *Npc1^nmf164^* mice/age).

Given the role played by microglia in the clearance of apoptotic cells, supernumerary synapses and myelin debris during cerebellum development (Arandjelovic & Ravichandran, 2015; Perez-Pouchoulen et al., 2015), we determined the abundance of microglia engaged in phagocytosis, as defined by the expression of specific markers and the presence of one or more phagocytic cups (cup-shaped invagination of the plasma membrane located at the tip of cell processes) (Perez-Pouchoulen et al., 2015; Vorselen et al., 2020) (Figure 2a). Our analysis revealed a significant increase of phagocytosis in the cerebellum of *Npc1^nmf164^* mice at P15, but not at P8 and P30, compared to *wt* mice (Figure 2b,c). Indeed, phagocytic microglia represent a significant fraction of the total microglia cells both in the anterior (11.6% ± 3.3) and posterior (18.7% ± 7.6) cerebellar lobules of *Npc1^nmf164^* mice at the end of the second postnatal week of age (Figure 2c - Phagocytic microglia). Consistently, the frequency of phagocytic cups was found significantly increased in cerebella of P15 *Npc1^nmf164^* (Figure 2c, phagocytic cups). To gain an insight on the material engulfed by microglia, we measured the diameter of phagocytic cups in the IGL of *wt* and *Npc1^nmf164^* P15 mice, observing no difference in the cup diameter between *wt* and *Npc1^nmf164^* mice (Figure 2c –Phagocytic cup size). Phagocytic cup diameter, 6.4 ± 0.6 μm for *wt* and 6.9 ± 0.4 μm for *Npc1^nmf164^*, is consistent with the engulfment of apoptotic bodies (Vorselen et al., 2020), while the concomitant engulfment of small cell debris and synapse by smaller phagocytic cups cannot be ruled out. To possibly associate the presence of apoptotic bodies to ongoing apoptosis we have determined the content of both pro-caspase 3 and cleaved caspase 3 in total protein extracts obtained from P8-30 *Npc1^nmf164^* and *wt* mouse cerebella, observing a marked difference between genotypes at P8 only (Supporting Information: Figure S3A,B).

Enhanced microglia clearance activity is typically sustained by improved recognition and internalization mechanisms and/or increased microglia surveillance and targeting of potential cargos (Sierra et al., 2013). To verify this possibility, we measured the expression of two well-established “eat-me” surface receptors, such as the Triggering receptor expressed on myeloid cells 2 (*Trem2*) and Cluster of differentiation molecule 11b (*Cd11b*), which are involved in the endocytosis of nonopsonic or opsonic targets, respectively (Sierra et al., 2013), along with the expression of Cluster of differentiation 68 (*Cd68*), a phagolysosomal marker, which is commonly used as a proxy of lysosomal activity. Our analysis showed that *Trem2* transcript expression undergoes a steady increase in P8-15 *Npc1^nmf164^* mouse cerebella, with a significant up-rise at P15 (Figure 2d). A similar up-rise of *Cd11b* transcripts was also observed in P15 *Npc1^nmf164^* mouse cerebella (Figure 2d), while a transient but significant reduction of *Cd68* transcripts was observed in *Npc1^nmf164^* mice at P15, in spite of its significant increase at P11 (Figure 2d). The upregulation of Trem2 expression in *Npc1^nmf164^* mice was also confirmed by Western blot analysis (Supporting Information: Figure S4).

### 3.3 The enhanced phagocytic activity of microglia is triggered by the parenchyma milieu of *Npc1^nmf164^* mice

**Figure 3:**
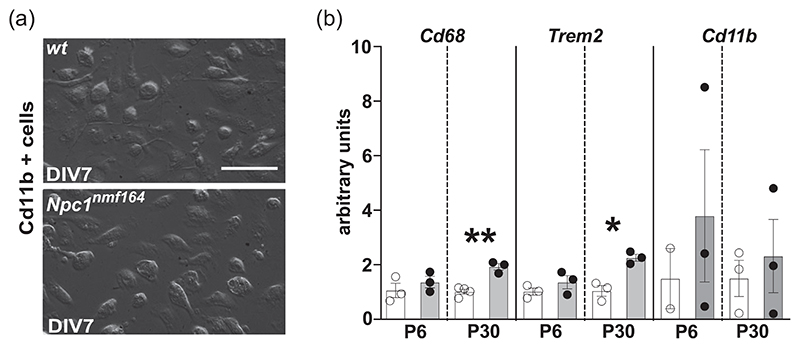
Gene expression analysis of isolated microglial cells from P6 and P30 *wt* and *Npc1^nmf164^* mice. (a) Representative images of in vitro primary microglia cultures obtained from *wt* and *Npc1^nmf164^* mice, 7 days after isolation. Scale bar: 50 μm. (b) RT-qPCR analysis of *Cd68*, *Trem2*, and *Cd11b* mRNA expression levels at the time points indicated. S16 expression was used for normalization. Empty bars: *wt*; gray filled bars: *Npc1^nmf164^*. Data are presented as mean ± SEM (Welch *t*-test, *p < 0.05, **p < 0.01; *n* = 2–4 *wt*, 3 *Npc1^nmf164^* mice/age).

The upregulation of the two markers of microglia activation and phagocytosis, Cd68 and Trem2, as early as at the second week of cerebellum development (P11, Figure 2d), prompted us to determine if increased phagocytosis was displayed by isolated microglial cells also. To this end, microglia cells were isolated from whole brains of *wt* and *Npc1^nmf164^* P6 and P30 mice, in vitro cultured for 7 days (DIV7) and processed for RT-qPCR analysis. We routinely obtained microglia cell counts in the approximate range of 200.000–800.000 cells/brain from both pups and adult mice (Figure 3, Supporting Information: Figure S5a), observing no difference in cell yields neither between genotype or age. The purity of isolated microglia cultures was confirmed by immunofluorescence using specific markers for microglia (Iba1), neurons (Tuj1), and astrocytes (GFAP) (Supporting Information: Figure S5b). Although the isolation procedure causes some changes of gene expression, after a few days in culture the expression of typical markers is readily established (Bohlen et al., 2017), making DIV7 microglia cultures a suitable model system for answering our question.

Our analysis revealed that microglia isolated from P6 *Npc1^nmf164^* pup cerebella did not show differences in the transcript level of either *Cd68* or *Trem2* compared to controls (Figure 3b). Conversely, microglia isolated from P30 *Npc1^nmf164^* mouse cerebellum showed a significant 2-fold increase of both *Cd68* and *Trem2* transcripts (Figure 3b). As for *Cd11b* expression, we found that it tended to increase on microglia isolated from both P6 and P30 *Npc1^nmf164^* mouse cerebella, but values displayed a very high variability (Figure 3b). In all, these findings suggest that changes on the expression patterns of microglial activation markers observed in *Npc1^nmf164^* mice, occur as a response to alterations in the surrounding microenvironment.

### 3.4 Microglia activation in the olfactory bulb of *Npc1^nmf164^* mice largely precedes sensory deficits

**Figure 4:**
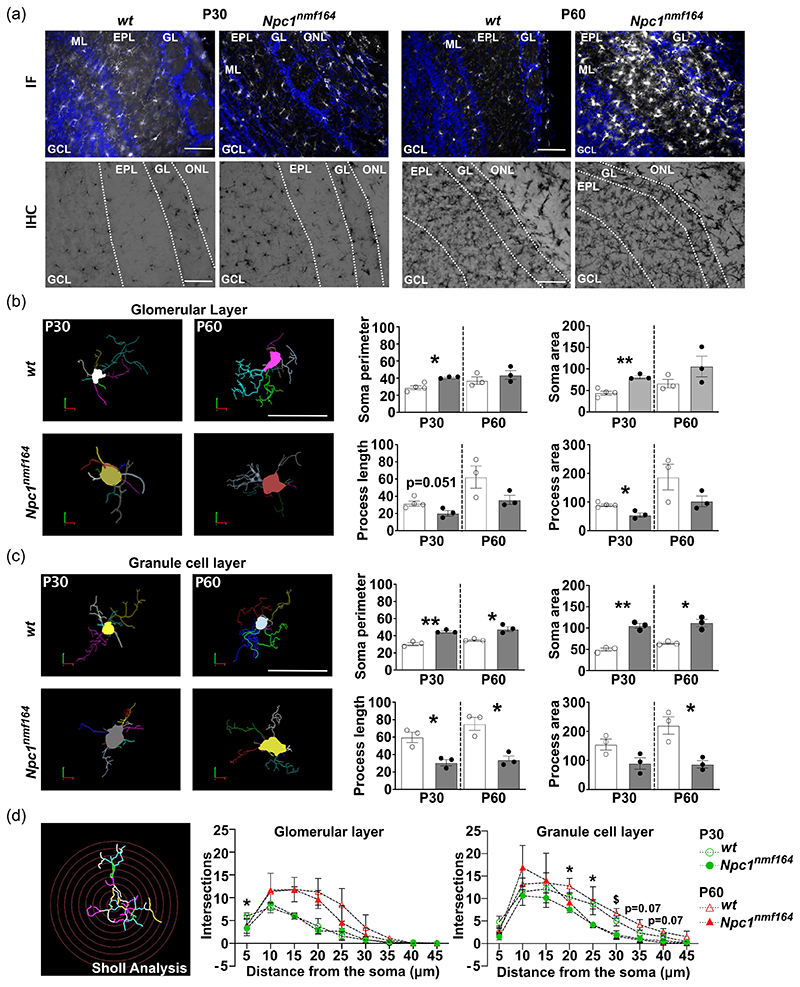


**Figure 6:**
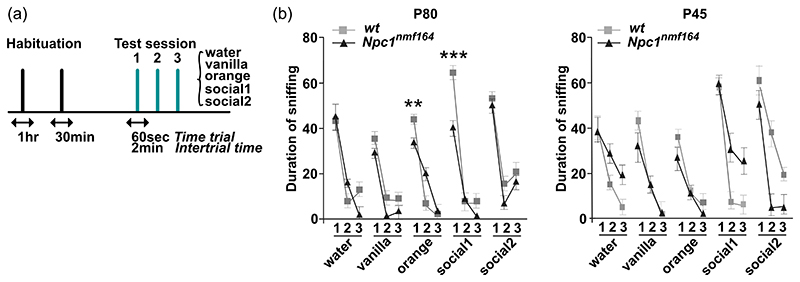
Performance of *wt* and *Npc1^nmf164^* mice in the habituation/dishabituation test. (a) Schematic representation of the habituation/dishabituation paradigm: the exploratory activity was determined by measuring the time (sec) that mice spent sniffing water (control), or odor-wet cotton swabs (the cotton tip part of the applicator). The cotton swab was freshly prepared for every exposure using a fixed amount of solution (5 and 4 μl for nonsocial and social odors, respectively). Each mouse was exposed to three consecutive 2 min presentations of each odor, according to the following order: water, nonsocial (vanilla, orange), and social (social 1 and social 2). The intertrial interval was 2 min, which is about the time needed to change the odor stimulus. (b, c) Assessment of the olfactory ability of P80 (b) and P45 (c) *Npc1^nmf164^* mice and age-matched *wt* controls by the habituation/dishabituation test. Data are presented as the mean ± SEM (statistical significance was determined using One-way analysis of variance followed by Bonferroni post-hoc, **p < 0.01, ***<0.001; *n* = 10 mice/age/genotype). Social 1: urine from unfamiliar mice of the opposite sex; social 2: urine from unfamiliar mice of same sex.

Besides the cerebellum (Caporali et al., 2016; Kavetsky et al., 2019), glial activation and defective neurogenesis have been reported to occur in the olfactory bulb of *Npc1*-/- mice and linked to sensory deficits at presymptomatic/symptomatic stages of the disease—4 and 8 weeks after birth (Dragotto et al., 2019; Hovakimyan et al., 2013; Meyer et al., 2018; Seo et al., 2014). To better trace the onset of microglia activation at the level of the olfactory bulb, we performed a detailed analysis of Iba1 immunoreactivity across the olfactory bulb of *wt* and *Npc1^nmf164^* juvenile mice. While no differences in the extent of microglia activation were detected at P15 (not shown), Iba1 immunoreactivity was significantly augmented in young adult Npc1-deficient mice when compared to *wt* starting from P30, as determined either by immunofluorescence or immunohistochemistry (Figure 4a). Microglia activation was prominent in the GCL and the GL indicated by the progressive decrease of exploration time when the same odor was reintroduced for the 2nd and 3rd time (habituation) and a reinstatement of the tendency to sniff for a longer time period when a novel odor was presented (Figure 6b). However, in comparison to *wt* mice, *Npc1^nmf164^* mice showed a significant decrease of exploration time for orange (*p* < 0.01**) and social 1 (*p* < 0.001***) (Figure 6b). This observation underscored the presence of a deficit in odor discrimination at a relatively early stage of the disease, the onset of which was traced by analyzing the performance of P45 *Npc1^nmf164^* mice in a similar paradigm. No significant differences were found between *Npc1^nmf164^* and *wt* mice, although the exploration time of *Npc1^nmf164^* mice was consistently shorter when they were challenged with a novel odor, with particular reference to nonsocial ones (Figure 6b).

**Figure 7:**
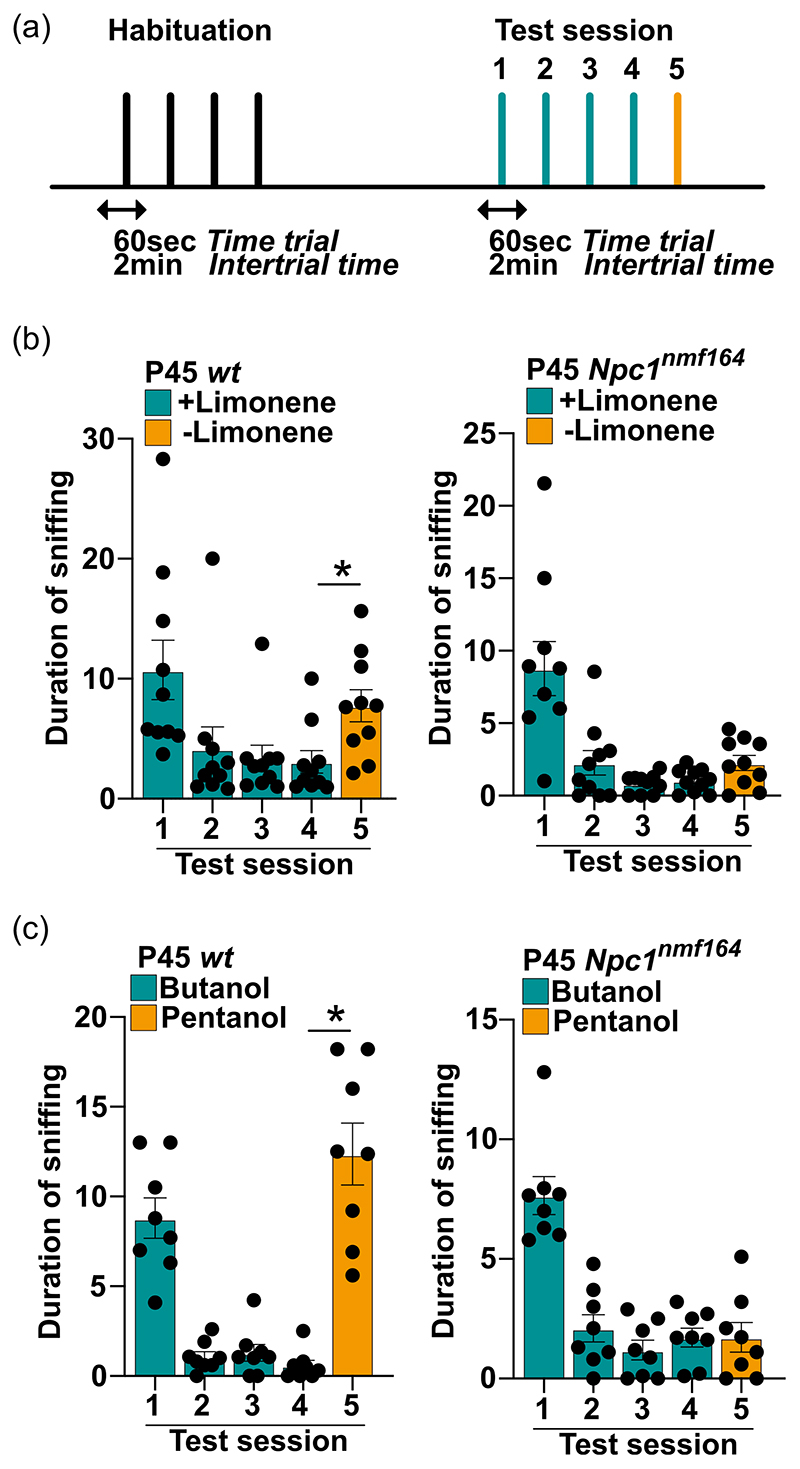
Performance of P45 *wt* and *Npc1^nmf164^* mice in the discrimination of enantiomeric pairs in a cotton tip presentation-based task. (a) A scheme of the odor discrimination paradigm: the ability to discriminate similar odors was assessed by measuring the time spent by *wt* and *Npc1^nmf164^* mice sniffing the structurally similar molecules, limonene (+) and limonene (-) or butanol and pentanol. Each mouse was exposed for 60 s to four consecutive presentations of one compound, while, in the last session, the structurally similar molecule was presented. The intertrial interval was 2 min, which is about the time needed to change the odor stimulus. (b, c) Assessment of the olfactory ability of P45 *Npc1^nmf164^* mice and age-matched *wt* controls in discriminating limonene (+)/limonene (-) (b) or butanol/pentanol (c) odors. Data are presented as mean ± SEM (statistical significance was determined using One-way analysis of variance followed by Bonferroni post-hoc tests, *p < 0.05; *n* = 10 mice/age/genotype).

This observation prompted us to further characterize fine olfaction abilities of *Npc1^nmf164^* mice, challenging them with nonsocial odors that share a similar chemical as detailed under Section 2. Thus, mice were subjected to four habituation trials in which (+)-Limonene was used as odorant, followed by a test trial in which the (–)-Limonene was used as novel odorant (Figure 7a). An exploration time of the novel, although structurally similar odorant (either (–)-Limonene or pentanol) longer than that measured at the fourth presentation of the first odorant (either [+]-Limonene or butanol) indicates that the animal is provided with fine olfactory discrimination ability (Abraham et al., 2010). In agreement with results of habituation/dishabituation test, *Npc1^nmf164^* mice displayed a habituation pattern similar to that of *wt* mice, as indicated by the progressive decrease of exploration time across repeated presentation (Figure 7b,c). However, when the (+)-Limonene-laced cotton swab was substituted with the (–)-Limonene-laced cotton swab (test session), a sharp and significant increase of the exploration time was detected with *wt* (*p* < 0.05*), but not *Npc1^nmf164^* mice (Figure 7b). A similar result was obtained when butanol and pentanol were used in the habituation and test session, respectively (Figure 7c).

Altogether, these findings indicate that *Npc1^nmf164^* mice show an impaired olfactory discrimination ability, especially in high challenging tasks, at early presymptomatic stages of the disease.

## 4 Discussion

Microglia cells show an extraordinary phenotypic and functional heterogeneity (Tan et al., 2020). Their density varies among brain areas, indicating that microglia migration, proliferation, and differentiation are finely controlled both during development and in the adulthood (Stowell et al., 2018; Tan et al., 2020). Individual microglia survey and act within a limited territory, implying that appropriate cell numbers and spatial distribution is necessary to guarantee proper neural circuit development and maintenance (Stowell et al., 2018).

Previous studies have shown that microglia activation and proliferation is detected in the thalamocortical system, basal ganglia and cerebellum of *Npc1*-/- mice of 2 and 3 weeks of age (Baudry et al., 2003; Pressey et al., 2012). In this study, we provide evidence of microglia activation in the white matter (WM) of cerebellum as early as 8 days after birth and a few days later in molecular/Purkinje/ internal granule cell layers (ML/PCL/IGL) of *Npc1^nmf164^* mice. Clearly, these data reinforce the view that microglia activation largely precedes overt neuronal involvement, opening the question on mechanisms responsible for such activation. We favor the idea that subtle changes of cerebellar and olfactory bulb microenvironments play a major role based on the following lines of thinking. First, microglia activation is particularly pronounced in the cerebellar WM, where the earliest signs of axonal degeneration are detected at P9 in *Npc1*-/- mice (Ong et al., 2001). Second, various anomalies of cerebellar morphogenesis have been described in *Npc1* mice (Boyle et al., 2020; Caporali et al., 2016). Third, microglia activation is first detected in the anterior lobules, recalling the antero-posterior graded progression of neurodegeneration, typically observed in the cerebellum of *Npc1* mice (Martin et al., 2019).

The regional heterogeneity of microglia density itself is particularly intriguing. For instance, the microglia number increase in the WM rather than in ML and PCL cerebellum sub-fields of *Npc1^nmf164^* mice, is particularly robust as early as P8 and sharply decreases thereafter. This finding is in agreement with the presence of hyper-proliferative microglia precursors residing in the cerebellar WM of P4 *Npc1^nmf164^* mice (Boyle et al., 2020). Meanwhile, it raises the question on what triggers microglia hyper-proliferation in this area at this early age. To answer this question, we recall that a fundamental neurogenic niche within the cerebellar WM produces virtually all GABAergic interneurons before P7 (Leto et al., 2008; Weisheit et al., 2006) and that the development and differentiation of these neurons is altered in Npc1 mice (Rabenstein et al., 2019; Zervas et al., 2001). Therefore, a derangement of developmental trajectories of this specific neuronal population might be the driving force of this time/area specific microglia proliferation. Alternatively, microglia hyperproliferation could be an intrinsic property of WM microglia. This possibility is consistent with the finding that Npc1 loss of function leads to mTORC1 over-activation (Castellano et al., 2017), which in turn stimulates the microglia proliferative activity, as recently demonstrated in a mouse model of epilepsy (Zhao et al., 2018). However, it is also true that cerebellar microglia have higher rates of basal turnover (Stowell et al., 2018) and show a high responsiveness to local perturbations compared to microglia elsewhere in the brain (Grabert et al., 2016; Stowell et al., 2018), favoring the idea that microglia proliferation reflects a secondary response to altered cellular environment (Caporali et al., 2016; Nusca et al., 2014; Vivas et al., 2019). Additionally, our finding that in the posterior cerebellar lobules of *Npc1^nmf164^* mice the increase of microglia density is delayed compared to the anterior ones, highlights a regional heterogeneity, which would be hardly explained by an exclusive cell-autonomous effect of Npc1 deficiency.

We show that cerebellar microglia of *Npc1^nmf164^* mice display a hypertrophic/bushy/ameboid phenotype (i.e., increased size of the cell body, thicker and shorter processes), typical of immature and/or activated state (Savage et al., 2019) by P15, which is concomitant with the defective histogenesis of both neurons and glial cells of these mice (Caporali et al., 2016). The engagement of activated microglia in the removal of apoptotic neurons and/or pruning of redundant or immature synapses by phagocytosis is indicated by the transient increase of phagocytic cups in the developing cerebellum of *Npc1^nmf164^* mice during the second postnatal week of age. This morphological signature is consistent with the significant upregulation of phagocytic markers at this age, including Cx3cr1, Trem2, and Cd11b. Particularly relevant is the overexpression of Trem2, which is considered an immunomodulatory receptor, regulating various aspects of microglia activities, including microglia survival and proliferation, chemotactic migration, and anti-inflammatory response (McQuade et al., 2020; Painter et al., 2015). Moreover, Trem2 has been recently shown to sustain the microglia glycolytic metabolism by stimulating the mTORC1 activation (Ulland et al., 2017). This Trem2 activity likely contributes to the increased energy requirement of proliferative and phagocytic microglia of *Npc1^nmf164^* mice.

The presence of apoptotic bodies inside phagocytic cups in sections of the cerebellar cortex of P14 *Npc1^nmf164^* was recently described (Boyle et al., 2020). Our estimate of phagocytic cup diameter of approximately 6–7 m is consistent with the size of apoptotic bodies (Vorselen et al., 2020). However, our data show no correlation between the upregulation of pro-caspase 3/cleaved caspase 3, which occurs at P8, and the appearance of phagocytic cups, which occurs at P15. Meanwhile, the up raise of pro-caspase 3/ cleaved caspase 3 in P8 mice coincides with the physiological peak of granule neuron apoptosis (Cheng et al., 2011) and the higher rate of apoptosis detected in *Npc1^nmf164^* mice is consistent with the derangement of developmental trajectories in the early postnatal cerebellum of *Npc1^nmf164^* (Canterini et al., 2017; Caporali et al., 2016; Nusca et al., 2014). The increased microglia phagocytic activity at P15 appears more likely related to synaptic changes and maturation of climbing and mossy fibers, which typically maximizes between P12 and P17 (Hashimoto & Kano, 2005). Indeed, the presence of an exaggerated CF-PCs synapse elimination in *Npc1*-defcient mice has been reported recently (Boyle et al., 2020; Colombo et al., 2021). Additional proof that microglia activation cannot be solely explained by cell-autonomous mechanisms is provided by our finding that transcript levels of *Trem2* and *Cd68* in microglia isolated from P6 *Npc1^nmf164^* pups and in vitro cultured up to DIV7 did not increase when compared to those of microglia isolated from age-matched *wt* pups. By contrast, microglia isolated from young adult P30 mice showed transcriptomic signatures coherent with an activated status, with a marked upregulation of both *Trem2* and *Cd68*. This finding is in agreement with the presence of lysosomal defects and significant accumulation of unesterified cholesterol in *Npc1*-deficient microglia largely in advance the appearance of signs of microglia activation (Colombo et al., 2021; Cougnoux et al., 2018). It also indicates that cholesterol accumulation is not sufficient to trigger microglia activation per se, but other activating signals from a dysfunctional microenvironment are necessary to induce significant and long-lasting microglia changes.

We observed similar changes of microglial cell density and morphology in the OB of *Npc1^nmf164^* mice. Hence, we report for the first time a robust increase of microglia phagocytic activity, which is particularly pronounced in the GCL and GL of *Npc1^nmf164^* mice starting from P30. This finding is consistent with previous studies, showing abnormal microgliosis in the OB of *Npc1*^-/-^ starting from the fourth week of age (Seo et al., 2014), when neurodegeneration is not present, yet, although subtle cellular alterations of peripheral components of the olfactory system are present (Hovakimyan et al., 2013). Thereafter, severe neuropathological changes in the OB and olfaction deficits are clearly seen in 8–10 weeks old *Npc1*^-/-^ mice (Hovakimyan et al., 2013; Seo et al., 2014; S. R. Yang et al., 2006). If one corrects for the more rapid progression of disease in this infantile model, the 8–10 weeks aged mice might be comparable at least to 90-day aged *Npc1^nmf164^* mice, supporting our conclusion that microglia activation in P30-60 *Npc1^nmf164^* can be considered an early sign of slight alterations of the olfactory system.

Clearly, this raises interest for the environmental perturbations that cause microglia activation in presymptomatic *Npc1^nmf164^* mice. Unfortunately, our knowledge of temporal-spatial cellular alterations of the OB of this disease mouse model is still limited. On the other hand, anomalies of the post-natal cerebellum of *Npc1^nmf164^* mice have been characterized with great details (reviewed in Fiorenza et al., 2022), making it possible to assume that for both areas the main trigger is a region-/time-specific derangement of neuronal differentiation and synaptic plasticity.

The unique regenerative nature of the OB and the subsequent continuous refinement of its circuitry make this area particularly vulnerable to the deficiency of Npc1 function. This is because both the generation of GCs, with particular reference to Calbindin-expressing periglomerular granules and the refinement of olfactory glomerular circuitry rely on Shh signaling (Angot et al., 2008; Persson et al., 2014), which is known to be altered in *Npc1* mice (Canterini et al., 2017; Formichi et al., 2018). GCs are rapidly integrated in the olfactory circuits where they modulate odor responsiveness and discrimination by shaping the activity of mitral and tufted cells within inhibitory microcircuits and allowing the fine-tuning of incoming sensory information processing (Abraham et al., 2010). The reduced ability to discriminate between highly similar odorants as early as at P45 suggests that a lower number of GCs had integrated properly in the pre-existing circuitry of the olfactory bulb of *Npc1^nmf164^* mice. Defective GC integration could begin early and persist throughout lifespan, causing limited odor response. This possibility is supported by our observation that the mislocalization of GCs actually disorganizes the cellular array of *Npc1^nmf164^* olfactory bulb.

There is also a second source of synaptic plasticity in the olfactory bulb that is provided by the continuous synaptogenesis between axonal projections of olfactory receptor neurons and dendrites of mitral/tufted cells at glomeruli (Lledo et al., 2005). These processes are expected to be deranged in *Npc1^nmf164^* mice as they are also regulated by the Shh signaling pathway (Persson et al., 2014), thus causing a general degeneration and a consequent impairment of all facets of olfactory functions, that is, odor detection threshold, odor identification and discrimination (Buschhueter et al., 2008).

Further studies are required to thoroughly characterize how microglia interplay with cellular alterations of both central and peripheral components of the olfactory system, increasing our knowledge of mechanisms that hit the detection of complex sensory stimuli in *Npc1^nmf164^* mice. As early deterioration of olfactory performance is a feature of many neurodegenerative diseases, including the most diffuse lysosomal storage Gaucher's disease (McNeill et al., 2012), the *Npc1^nmf164^* mouse model might represent a valuable model system to gain an insight on mechanisms affecting olfaction in a larger family of neurodegenerative diseases.

## Supplementary Material

Figure S1

Figure S2

Figure S3

Figure S4

Figure S5

Figure S6

Figure S7

## Figures and Tables

**Figure 5:**
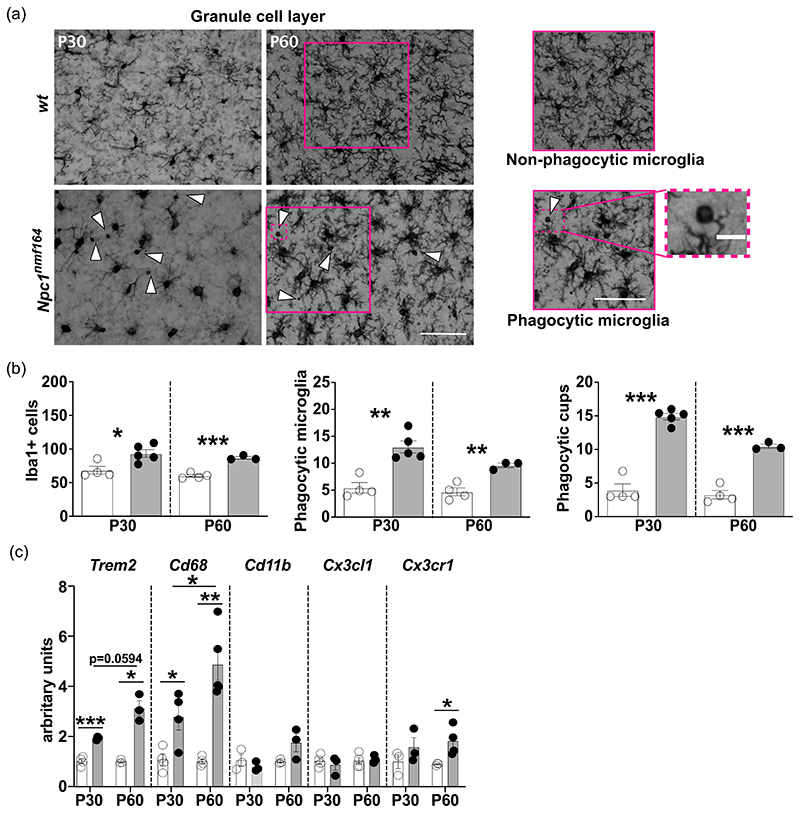
Microglia in the olfactory bulb of *Npc1^nmf164^* mice display enhanced phagocytic activity. (a) Representative images (left to right) of GCL microglia of P30 and P60 *wt* and *Npc1^nmf164^* mice. Arrowheads indicate Iba1-positive cells provided with phagocytic cups. Scale bars: 50 μm. Higher magnification of nonphagocytic and phagocytic microglia, with inset showing phagocytic cups (Scale bar: 8 μm). (b) Quantitative analysis of the fraction of Iba1-positive microglia (left), phagocytic microglia (center) and total number of phagocytic cups in 500 μm^2^ ROI (right) in the GCL of P30 and P60 *wt* and *Npc1^nmf164^* mice. Empty bars: *wt*; gray filled bars: *Npc1^nmf164^* mice. Data are presented as mean ± SEM (Welch *t*-test, *p < 0.05, **<0.01, ***<0.001; *n* = 4 *wt*, *n* = 3–5 *Npc1^nmf164^* mice/age). (c) RT-qPCR analysis of mRNA expression of genes associated with activated/phagocytic microglia in olfactory bulbs collected from P30 and P60 *wt* and *Npc1^nmf164^*. S16 expression was used for normalization. Empty bars: *wt*; gray filled bars: *Npc1^nmf164^*. Data are presented as mean ± SEM (Welch *t*-test, *p < 0.05, **<0.01, ***<0.001; *n* = 3–4 *wt*, *n* = 3–5 *Npc1^nmf164^* mice/age).

## Data Availability

Data supporting present findings are available from the corresponding author upon reasonable request.
