# Supplementary material for: The appearance of phagocytic microglia in the postnatal brain of Niemann Pick type C mice is developmentally regulated and underscores shortfalls in fine odor discrimination
Source: J Cell Physiol. 2022 Nov 2;237(12):4563–79. doi: 10.1002/jcp.30909 (PMC7613956; doi:10.1002/jcp.30909)
Supplement: Supplementary file 1 — Supporting information. [file JCP-237-4563-s001.pdf]

a

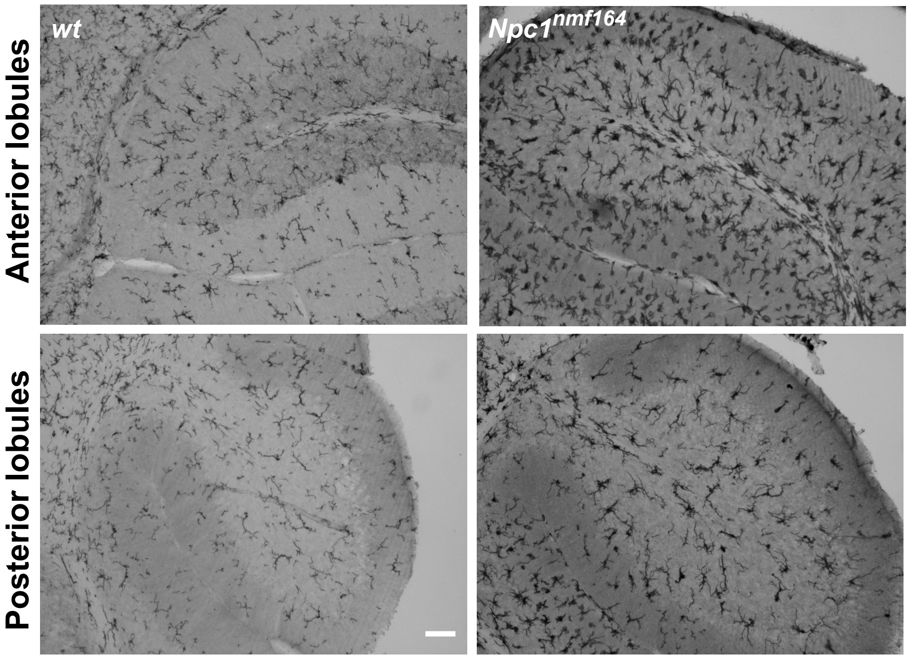

Figure S1. Robust microglia activation in the cerebellum of P60 *Npc1^{nmf164}* mice. (a, b) Representative images of Iba1-positive microglia in the anterior (II) and posterior (X) lobules of *wt* and *Npc1^{nmf164}* mice. Scale bars: 50 µm (n = 3 *wt*, 2 *Npc1^{nmf164}* mice).
