# Supplementary material for: The appearance of phagocytic microglia in the postnatal brain of Niemann Pick type C mice is developmentally regulated and underscores shortfalls in fine odor discrimination
Source: J Cell Physiol. 2022 Nov 2;237(12):4563–79. doi: 10.1002/jcp.30909 (PMC7613956; doi:10.1002/jcp.30909)
Supplement: Supplementary file 2 — Supporting information. [file JCP-237-4563-s003.pdf]

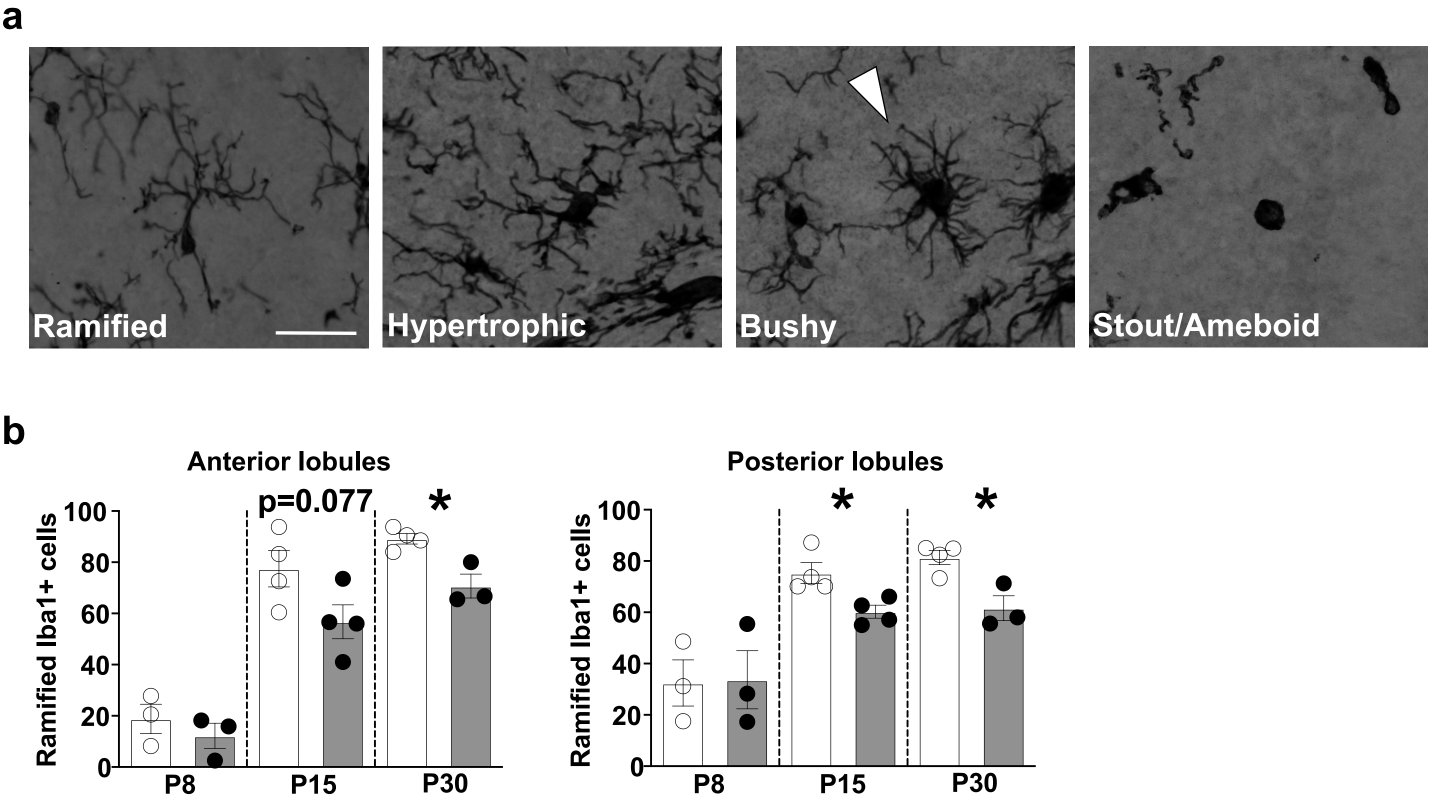

Figure S2. Morphological changes of microglia in the developing cerebellum of *wt* and *Npc1*^{nmf164} mice. (a) Representative images of the distinctive morphological phenotype of Iba1-positive cerebellar microglia (b) Bars indicate the fraction of Iba1-positive cells with ramified morphology in the anterior and posterior cerebellar lobules. Empty bars: *wt*; grey filled bars: *Npc1*^{nmf164}. Data are presented as mean ± SEM (Welch T-test, * p<0.05; n= 3-4 *wt*, 3-4 *Npc1*^{nmf164} mice/age).
