# Supplementary material for: The appearance of phagocytic microglia in the postnatal brain of Niemann Pick type C mice is developmentally regulated and underscores shortfalls in fine odor discrimination
Source: J Cell Physiol. 2022 Nov 2;237(12):4563–79. doi: 10.1002/jcp.30909 (PMC7613956; doi:10.1002/jcp.30909)
Supplement: Supplementary file 3 — Supporting information. [file JCP-237-4563-s006.pdf]

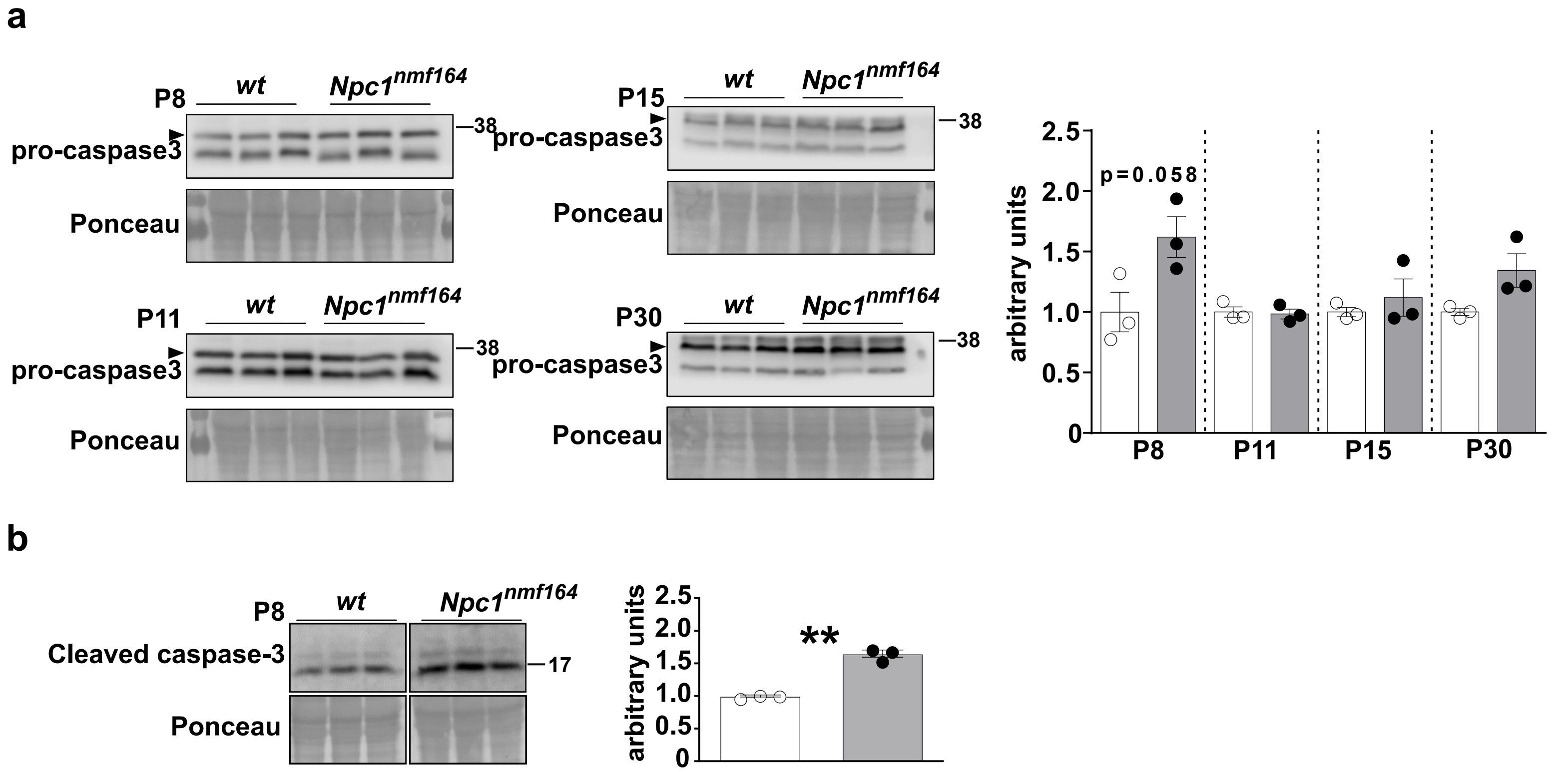

Figure S3. Apoptosis marker expression in P8-P30 cerebella. (a) Representative Western blots and relative quantification (bars on the right; empty bars: *wt*; grey filled bars: *Npc1*^{nmf164}) of pro-caspase3 protein expression in cerebella of P8-P30 *wt* and *Npc1*^{nmf164} mice. Arrowheads indicate bands of interest. For each experiment, densitometric values were normalized to the total protein content. (b) Representative Western blots and relative quantification (bars on the right) of cleaved caspase-3 in cerebella of P8 *wt* and *Npc1*^{nmf164} mice. Empty bars: *wt*; grey filled bars: *Npc1*^{nmf164}. Data are presented as mean ± SEM (Welch T-test, ** $p<0.01$; n = 3 *wt*, 3 *Npc1*^{nmf164}).
