# Supplementary material for: The appearance of phagocytic microglia in the postnatal brain of Niemann Pick type C mice is developmentally regulated and underscores shortfalls in fine odor discrimination
Source: J Cell Physiol. 2022 Nov 2;237(12):4563–79. doi: 10.1002/jcp.30909 (PMC7613956; doi:10.1002/jcp.30909)
Supplement: Supplementary file 4 — Supporting information. [file JCP-237-4563-s004.pdf]

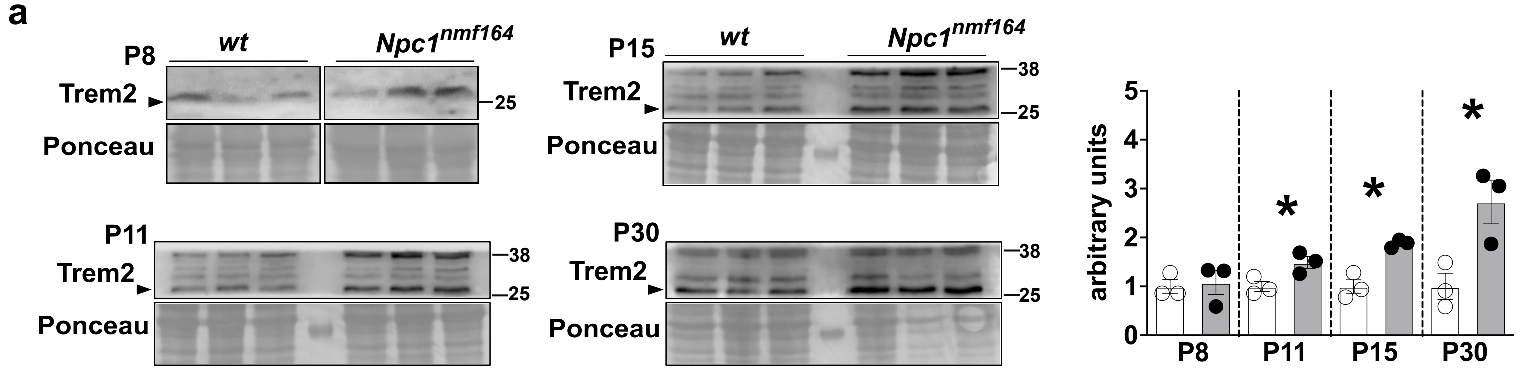

Figure S4. Trem2 protein expression increases in the cerebellum of P15 *Npc1[nmf164]* mice. (a) Representative Western blots and relative quantification (bars on the right) of Trem2 protein expression in the cerebellum of P8-P30 *wt* and *Npc1[nmf164]* mice. For each experiment, densitometric values were normalized to the total protein content. Arrowheads indicate bands of interest. Empty bars: *wt*; grey filled bars: *Npc1[nmf164]*. Data are presented as mean ± SEM (Welch T-test, * p<0.05; (n = 3 *wt*, 3 *Npc1[nmf164]* mice/age).
