# Supplementary material for: The appearance of phagocytic microglia in the postnatal brain of Niemann Pick type C mice is developmentally regulated and underscores shortfalls in fine odor discrimination
Source: J Cell Physiol. 2022 Nov 2;237(12):4563–79. doi: 10.1002/jcp.30909 (PMC7613956; doi:10.1002/jcp.30909)
Supplement: Supplementary file 5 — Supporting information. [file JCP-237-4563-s007.pdf]

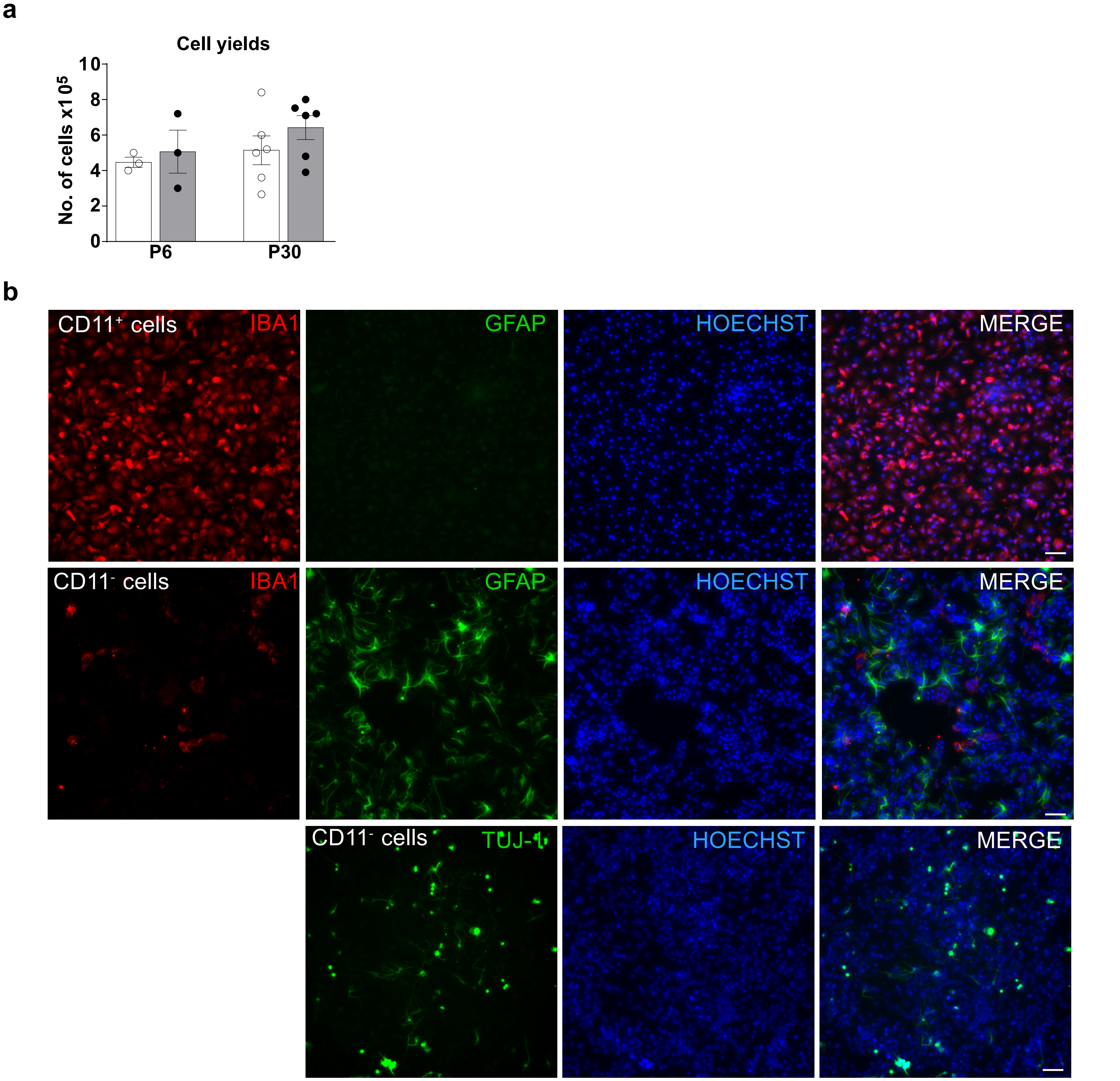

Figure S5. Characterization of microglia isolated from *wt* and *Npc1*^{nmf164} mice. (a) Determination of Cd11b+ cells yields from brains of P6 and P30 *wt* and *Npc1*^{nmf164} mice by haemocytometer counting. Empty bars: *wt*; grey filled bars: *Npc1*^{nmf164}. (b) Representative images of immunofluorescences performed on CD11b+ and CD11b- fractions for lineage-specific markers: microglia (Iba1, 1:100, Wako, red), neurons (Tuj1, 1:100 Cell Signalling, green) and astrocytes (GFAP, 1:50, Santa Cruz, green). Nuclei were stained with Hoechst (1:1000, Hoechst 33258, Sigma-Aldrich, blue).
