# Supplementary material for: The appearance of phagocytic microglia in the postnatal brain of Niemann Pick type C mice is developmentally regulated and underscores shortfalls in fine odor discrimination
Source: J Cell Physiol. 2022 Nov 2;237(12):4563–79. doi: 10.1002/jcp.30909 (PMC7613956; doi:10.1002/jcp.30909)
Supplement: Supplementary file 6 — Supporting information. [file JCP-237-4563-s008.pdf]

A

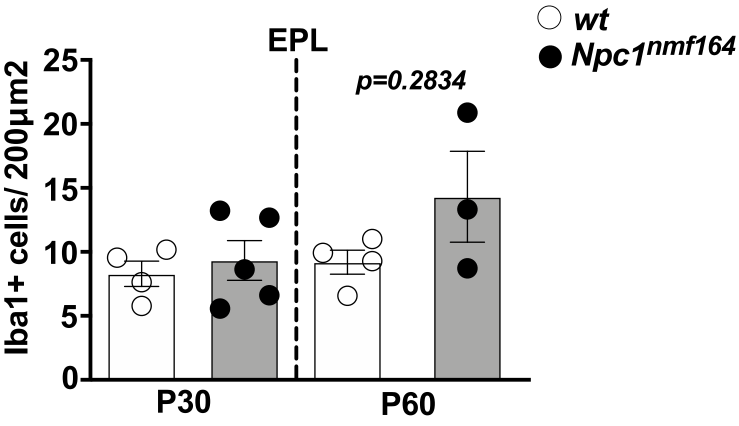

Figure S6. Quantitative analysis of the fraction of Iba1-positive cells in the EPL of P30 and P60 *wt* and *Npc1*^{nmf164} mice (ROI: 200 µm^2). Empty bars: *wt*; grey filled bars: *Npc1*^{nmf164}. Data are presented as mean ± SEM (Welch T-test, n = 4 *wt*, 3-5 *Npc1*^{nmf164} mice/age).
