# Supplementary material for: The appearance of phagocytic microglia in the postnatal brain of Niemann Pick type C mice is developmentally regulated and underscores shortfalls in fine odor discrimination
Source: J Cell Physiol. 2022 Nov 2;237(12):4563–79. doi: 10.1002/jcp.30909 (PMC7613956; doi:10.1002/jcp.30909)
Supplement: Supplementary file 7 — Supporting information. [file JCP-237-4563-s005.pdf]

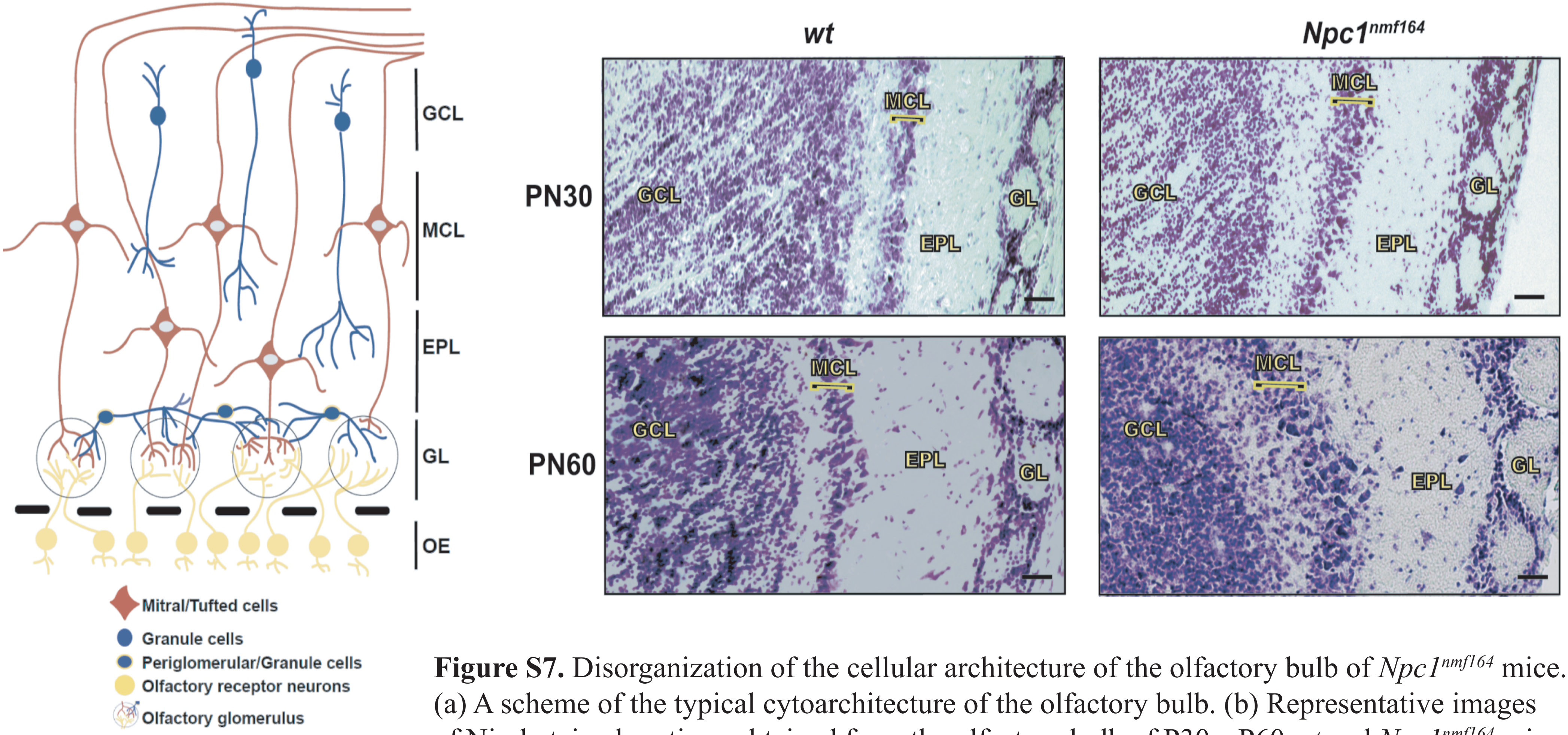


**Figure S7.** Disorganization of the cellular architecture of the olfactory bulb of *Npc1*$^{nmf164}$ mice. (a) A scheme of the typical cytoarchitecture of the olfactory bulb. (b) Representative images of Nissl-stained sections obtained from the olfactory bulb of P30 e P60 *wt* and *Npc1*$^{nmf164}$ mice (n=3 wt, 4 *Npc1*$^{nmf164}$ mice/age). Scale bar: 80 µm. GCL: granule cell layer; EPL: external plexiform layer; GL: glomerular layer; OE: olfactory epithelium.
