# Supplementary material for: The appearance of phagocytic microglia in the postnatal brain of Niemann Pick type C mice is developmentally regulated and underscores shortfalls in fine odor discrimination
Source: J Cell Physiol. 2022 Nov 2;237(12):4563–79. doi: 10.1002/jcp.30909 (PMC7613956; doi:10.1002/jcp.30909)
Supplement: Supplementary file 8 — Supporting information. [file JCP-237-4563-s002.pdf]

Table 1S: Morphometry of 3D reconstructed Iba1-positive cells from olfactory glomerular cell layer (GL)			
**P30**	***wt*** (n=20 cells/4 mice)	***Npc1***^{nmf164} (n=17 cells/3 mice)	**Statistics**
**Parameters**	**Mean ± SD**	**Mean ± SD**	**p-value**
Soma perimeter	28.81 ± 4.53	40.29 ± 1.15	**0.011***
Soma Area	43.99 ± 9.28	80.56 ± 6.10	**0.0015****
Max feret diameter	10.58 ± 1.82	14.07 ± 0.81	**0.024***
Min feret diameter	6.20 ± 0.57	8.38 ± 0.18	**0.0026****
Aspect ratio	1.75 ± 0.14	1.74 ± 0.10	0.923
Form factor	0.68 ± 0.07	0.63 ± 0.04	0.241
Solidity	0.92 ± 0.03	0.89 ± 0.02	0.225
Roundness	0.53 ± 0.07	0.53 ± 0.04	0.955
Compactness	0.72 ± 0.05	0.72 ± 0.03	0.91
Convexity	0.94 ± 0.01	0.92 ± 0.02	0.221
Processes	6.5 ± 1.01	8.54 ± 0.83	**0.033***
Nodes	16.33 ± 2.79	14.12 ± 3.54	0.425
Ends	25.27 ± 4.03	25.43 ± 3.12	0.954
Process length	191.3 ± 26.93	157.6 ± 21.84	0.128
Mean process length	31.50 ± 5.98	20.12 ± 5.48	0.051
Process surface	549.50 ± 98.07	425.90 ± 49.41	0.086
Mean process surface	88.42 ± 8.19	54.32 ± 13.69	**0.030***
Process volume	131.4 ± 40.53	93.96 ± 8.52	0.161
Mean process volume	20.56 ± 2.65	11.98 ± 2.76	**0.012***
**P60**	***wt*** (n=12 cells/3 mice)	***Npc1***^{nmf164} (n=10 cells/3 mice)	**Statistics**
**Parameters**	**Mean ± SD**	**Mean ± SD**	**p-value**
Soma perimeter	37.08 ± 7.55	43.49 ± 8.90	0.397
Soma Area	65.98 ± 17.21	105.8 ± 41.49	0.233
Max feret diameter	13.21 ± 2.18	14.70 ± 3.23	0.548
Min feret diameter	7.74 ± 1.34	10.16 ± 1.66	0.124
Aspect ratio	1.75 ± 0.18	1.49 ± 0.21	0.182
Form factor	0.63 ± 0.08	0.68 ± 0.03	0.358
Solidity	0.90 ± 0.05	0.93 ± 0.00	0.43
Roundness	0.50 ± 0.05	0.62 ± 0.04	**0.043***
Compactness	0.70 ± 0.04	0.79 ± 0.03	**0.041***
Convexity	0.92 ± 0.03	0.91 ± 0.006	0.74
Processes	8.56 ± 2.67	10.08 ± 1.99	0.474
Nodes	39.06 ± 9.50	28.78 ± 3.67	0.193
Ends	53.28 ± 8.55	44.64 ± 3.86	0.216
Process length	407.00 ± 62.92	332.90 ± 49.58	0.188
Mean process length	62.32 ± 22.38	35.83 ± 9.03	0.166
Process surface	1197 ± 315.10	941.3 ± 148.00	0.297
Mean process surface	184.50 ± 81.39	102.40 ± 32.03	0.216
Process volume	292.60 ± 111.40	239.80 ± 40.24	0.506
Mean process volume	46.00 ± 23.22	25.81 ± 7.55	0.268

Table 2S: Morphometry of 3D reconstructed Iba1-positive cells from olfactory granule cell layer (GCL)			
**P30**	***wt*** (n=24 cells/3 mice)	***Npc1*** ^{nmf164} (n=27 cells/3 mice)	**Statistics**
**Parameters**	**Mean ± SD**	**Mean ± SD**	**p-value**
Soma perimeter	30.15 ± 2.95	44.48 ± 1.76	**0.004****
Soma Area	49.12 ± 6.46	105.10 ± 9.45	**0.0018****
Max feret diameter	10.90 ± 1.20	16.17 ± 0.72	**0.005****
Min feret diameter	6.68 ± 0.54	9.63 ± 0.71	**0.006****
Aspect ratio	1.66 ± 0.12	1.70 ± 0.053	0.611
Form factor	0.69 ± 0.052	0.72 ± 0.025	0.484
Solidity	0.91 ± 0.037	0.91 ± 0.006	>0.99
Roundness	0.56 ± 0.06	0.56 ± 0.006	0.93
Compactness	0.74 ± 0.046	0.74 ± 0.006	0.9
Convexity	0.95 ± 0.005	0. 95 ± 0.01	>0.99
Processes	8.33 ± 0.66	10.46 ± 2.64	0.295
Nodes	28.37 ± 3.73	19.14 ± 7.91	0.17
Ends	39.52 ± 3.53	30.84 ± 11.16	0.309
Process length	424.50 ± 40.28	286.5 ± 64.49	**0.044***
Mean process length	59.77 ± 10.33	30.72 ± 6.15	**0.021***
Process surface	1086.00 ± 152.90	808.3 ± 206.70	0.141
Mean process surface	154.60 ± 32.78	89.45 ± 34.12	0.076
Process volume	230.10 ± 43.45	203.0 ± 77.70	0.633
Mean process volume	33.10 ± 8.64	23.18 ± 13.36	0.351
**P60**	***wt*** (n=17 cells/3 mice)	***Npc1*** ^{nmf164} (n=18 cells/3 mice)	**Statistics**
**Parameters**	**Mean ± SD**	**Mean ± SD**	**p-value**
Soma perimeter	34.51 ± 1.37	47.74 ± 4.98	**0.036***
Soma Area	64.37 ± 3.82	111.80 ± 14.74	**0.025***
Max feret diameter	11.98 ± 0.39	14.71 ± 0.96	**0.026***
Min feret diameter	7.92 ± 0.48	10.84 ± 1.08	**0.028***
Aspect ratio	1.53 ± 0.10	1.37 ± 0.12	0.17
Form factor	0.69 ± 0.005	0.72 ± 0.12	0.77
Solidity	0.92 ± 0.006	0.93 ± 0.04	0.801
Roundness	0.58 ± 0.04	0.66 ± 0.07	0.179
Compactness	0.76 ± 0.03	0.81 ± 0.04	0.186
Convexity	0.93 ± 0.02	0.92 ± 0.04	0.74
Processes	7.84 ± 1.39	12.48 ± 2.22	**0.047***
Nodes	43.57 ± 9.46	32.57 ± 11.57	0.274
Ends	60.76 ± 11.46	52.00 ± 16.38	0.495
Process length	510.8 ± 46.33	382.6 ± 133.00	0.231
Mean process length	75.12 ± 12.82	33.76 ± 7.72	**0.014***
Process surface	1470.00 ± 199.60	987.90 ± 374.90	0.143
Mean process surface	219.90 ± 51.77	86.36 ± 21.91	**0.032***
Process volume	349.30 ± 75.72	208.4 ± 83.75	0.097
Mean process volume	53.42 ± 17.57	18.05 ± 4.95	0.064
